# Female Dominance over Males in Primates: Self-Organisation and Sexual Dimorphism

**DOI:** 10.1371/journal.pone.0002678

**Published:** 2008-07-16

**Authors:** Charlotte K. Hemelrijk, Jan Wantia, Karin Isler

**Affiliations:** 1 Theoretical Biology, Centre for Ecological and Evolutionary Studies, University of Groningen, Haren, The Netherlands; 2 Department of Informatics, University of Zurich, Zurich, Switzerland; 3 Anthropological Institute & Museum, University of Zurich, Zurich, Switzerland; Georgia State University, United States of America

## Abstract

The processes that underlie the formation of the dominance hierarchy in a group are since long under debate. Models of self-organisation suggest that dominance hierarchies develop by the self-reinforcing effects of winning and losing fights (the so-called winner-loser effect), but according to ‘the prior attribute hypothesis’, dominance hierarchies develop from pre-existing individual differences, such as in body mass. In the present paper, we investigate the relevance of each of these two theories for the degree of female dominance over males. We investigate this in a correlative study in which we compare female dominance between groups of 22 species throughout the primate order. In our study female dominance may range from 0 (no female dominance) to 1 (complete female dominance). As regards ‘the prior attribute hypothesis’, we expected a negative correlation between female dominance over males and species-specific sexual dimorphism in body mass. However, to our surprise we found none (we use the method of independent contrasts). Instead, we confirm the self-organisation hypothesis: our model based on the winner-loser effect predicts that female dominance over males increases with the percentage of males in the group. We confirm this pattern at several levels in empirical data (among groups of a single species and between species of the same genus and of different ones). Since the winner-loser effect has been shown to work in many taxa including humans, these results may have broad implications.

## Introduction

Dominance hierarchies based on agonistic interactions are observed in many group-living animals and in humans [1]. Their causation is under debate. According to the so-called ‘prior attribute hypothesis’ dominance hierarchies are supposed to arise from intrinsic attributes which are pre-existing individual differences in strength, such as body mass, age, sex or physiological traits [1]–[7]. According to the self-organisation hypothesis based on several models, a dominance hierarchy emerges in a group of individuals even in the absence of any pre-existing differences, through the self-reinforcing effects of winning and losing fights [4]–[13]. This so-called winner-loser effect implies that, after losing, an individual is more likely to lose again and vice versa after winning [14], [15]. There is much empirical evidence for the winner-loser effect: It appears to operate in many species, ranging from insects, crustaceans, fishes, amphibians, and reptiles, to mammals, including nonhuman primates [16]–[18] and even humans [for a recent review see,19]. Fighting experience alters an individual's fighting ability either because after a contest its ‘actual’ fighting ability is changed due to a neuroendocrine effect or because an individual has changed its perception of its own fighting ability. As to neuroendocrine changes, it is mainly corticosteroid and androgen titres that have been studied. In individuals that have recently lost, levels of corticosteroids are often increased and plasma testosterone levels are reduced [20], but the effects of winning experiences are less clear [19].

However, evidence for the winner-loser effect concerns mainly dyadic settings: in a group it appears to be difficult to distinguish between the contribution of the winner-loser effect and that of prior attributes to the dominance hierarchy [4]. Yet, there are some indications for the contribution of the winner-loser effect and self-organisation in a group, because the dominance relations between two individuals differ between different social contexts, namely being kept in a pair or in a group [while the pre-existing differences were similar for both contexts, 5], [21]. Similar differences are also found when dominance relations between two types of individuals are compared: compared to the dyadic setting the group context reduces relative dominance of the red over the blue morph of cichlids [22] and of bold individuals over cautious ones in great tits [23], [24]. In our earlier study we explain this reduction of dominance of red versus blue and of bold versus shy individuals in a group, by the greater loser effects these individuals suffer, because in a group they also fight with their own type, which does not happen when they are kept in a mixed pair [25].

From these results, we expect a similar effect for the relative dominance of females over males in primates. Since it is known that the degree of female dominance differs between groups of a single species (for unknown reasons) in many species, - for instance, in certain lemur species [26]–[29], bonobos [30]–[33], macaques, vervets, squirrel monkeys, and talapoins [34], - we infer that the contribution of self-organisation to it may be revealed by comparing the degree of female dominance between groups that differ in their group composition (i.e sex ratio). We use a model of the winner-loser effect to deduce predictions about the degree of female dominance in groups of different compositions, and test these predictions in empirical data. Furthermore, we investigate the evidence for the prior attribute hypothesis. Here, we expect that, in general, female dominance over males is greater in species with a weaker male-biased sexual dimorphism in body mass. We have tested both predictions in data of 22 primate species throughout the primate order.

Note that this is a new approach to the study of female dominance. So far the study of female dominance has been confined mainly to ecological and evolutionary hypotheses, such as those concerned with energy [e.g. see 26], [35] or sexual selection [36], and systematic studies have mainly been confined to the lemuriformes [37] or even only to those species where female dominance is complete [29]. In the present paper, female dominance indicates the dominance ranks of all females relative to those of all males in the group. This is calculated by means of a standardized Mann Whitney U-test, namely the Mann Whitney U-value is divided by its maximum value for the specific group size and sex ratio [38]. Its value ranges from 0 for complete female subordinance to all males, via a half for co-dominance with males, to 1 for complete female dominance over all males. Although the differences are usually small, this measure is preferable to the win-ratio of females over males, - which has been applied so far by others [26], [29], [39]- , because it takes into account the dominance position of all group members, whereas the win-ratio only concerns the frequency of winning between the sexes (not their relative dominance positions) and may give too much weight to certain dyads [40]. Our definition of the dominance rank of each individual is relative to that of others: The higher the percentage of winning of an individual from each of its interaction partners is on average, the higher also is an individuals' dominance rank [41].

Following the self-organisation hypothesis, we predict that female dominance may depend on group composition via self-organisation. To determine the specific effects of group composition on female dominance, we use an earlier model, because its results were firmly in accordance with empirical data of primate societies. In this model, called DomWorld [11], [12], [25], [38], the actions of individuals are restricted to grouping and competing, while the effects of winning and losing fights are self-reinforcing. These effects are smaller in those cases in which the outcome of a fight was expected (the lower ranking one loses) and greater if the outcome was unexpected (when the lower ranking was winning). The probability to win a fight depends on the fighting capacity of an individual relative to its opponent and also on chance. Groups with a high intensity of aggression (as testified by biting) appear in the model to resemble in many respects groups of intensely aggressive despotic macaques, whereas groups with low aggression-intensity (slaps and threats instead of biting) are similar to those of mild species with an egalitarian dominance style [11]. Dominance of females over males in the model appears to be greater in groups with a high intensity of aggression, and similar effects are found for high frequency of aggression [42]. This corresponds with an observation by Thierry that adolescent males are later in reaching dominance over females in tonkean macaques than in the despotic rhesus macaque [43], because their aggression is more intense; it also agrees with the finding that female dominance is greater among bonobos than among common chimpanzees [30], which is to be expected since the frequency of aggression is probably higher in bonobos because of their denser grouping [44]; however, these suggestions need further empirical verification.

To test our predictions, we use matrices of winning and of aggression taken from a broad survey of the available literature. We study the correlations at three levels: between species throughout the primate order (using the independent contrast method), between groups of species that are related (of the genus *Macaca*) and between groups of a single species (for *Macaca arctoides* and *M. mulatta*).

Our aim is to investigate whether female dominance over males may be due to sexual dimorphism, or to self-organisation or to these two combined. Our results indicate that the degree of female dominance over males depends on group composition rather than on effects of sexual dimorphism. This supports our hypothesis based on self-organisation.

## Results

### The model

**Figure 1:**
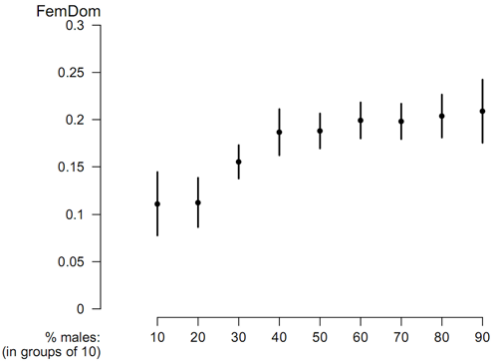
Mean and standard error of female dominance over males (FemDom) for different percentages of males in the group in the model. Intensity of aggression (StepDom) of males = 1, of females = 0.1, initial dominance of females and males is 16 and 32, respectively.

At a high intensity of aggression female dominance over males increases significantly with the percentage of males in the group (Figure 1, Table 1) but only when males start with a higher initial dominance value than the females and if there is a great difference in intensity of aggression between the sexes (*i.e*. if females had 10% of the aggression of males, not 80%). Apart from this, the correlation appears robust for the degree of sexual dimorphism in initial dominance values. (For this we tested initial dominance values of males versus females of 28 versus 20, 32 versus 16 and of 36 versus 12, respectively for sex ratios with 2, 4 and 6 males in groups of 12 (data not shown). We did not go above 50% males in the group, because this is rare in nature.) At a low intensity of aggression, this correlation is absent [for more detailed studies of these effects see Wantia and Hemelrijk (in prep), 36].

**Table 1:** Kendall correlation (Tau) between female dominance and the proportion of males in a group for different initial dominance values and intensities of aggression (average of 40 runs).

Initial DomValues (m , f)	Intensity of aggression (m , f)		Tau	P
32 , 16	High	(1 , 0.8)	0.028	NS
		(1 , 0.1)	0.944	***
	Low	(0.1 , 0.08)	n.a.	n.a.
		(0.1 , 0.01)	0.117	NS
24 , 24	High	(1 , 0.8)	0.444	NS
		(1 , 0.1)	0	NS
	Low	(0.1 , 0.08)	0.140	NS
		(0.1 , 0.01)	0.277	NS

m = male, f = female. N = 9 sex ratios. NS = ‘not significant’, ^***^ = p<0.001 two-tailed, n.a. = not available.

### Discussion

In our model female dominance increases with the percentage of males, but only if there is sufficient difference in intensity of aggression between the sexes. In that case (and only in that case) a higher number of males leads not only to a higher number of interactions with males (N = 360, Tau = 0.148, P = 0.0001), but also to a sufficiently higher average intensity of aggressive interactions, so that more female dominance develops via a stronger hierarchical differentiation per sex [11], [12], [38]. Simultaneously, this leads to a society that resembles the society of despotic macaques [11], [38]. At a low aggression intensity (for societies that resemble egalitarian macaques), the correlation with female dominance is lacking, because females remain mostly completely subordinate to males even in groups with a higher percentage of males. This is the result of the low impact of each interaction and the absence of a social-spatial structure. This difference in effect of high and low intensity of aggression is only a quantitative one, not a qualitative one, since both correlations are positive (Table 1).

In sum, following the hypothesis of self-organisation, we expected to find in empirical data that female dominance over males increases with the percentage of males in the group.

### Empirical Data

We investigated whether female dominance relative to males increases with (a) the percentage of males in the group, or (b) the degree of sexual dimorphism, or with both.

**Figure 2:**
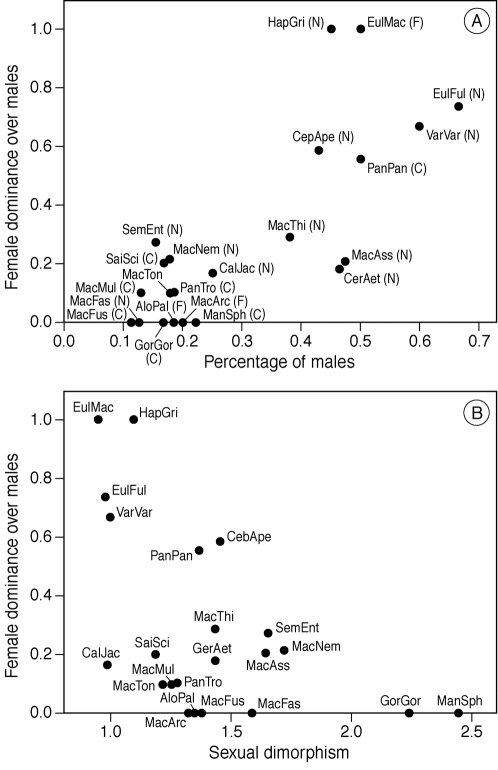
a) Female dominance and the proportion of males in a group (medians). b) Female dominance and male biased sexual dimorphism (weight male/ weight female). AloPal = *Alouatta palliata*; CalJac = *Callithrix jacchus*; CebApe = *Cebus apella*; CerAet = *Cercopithecus aethiops*; EulFul = *Eulemur fulvus rufus*; EulMac = *Eulemur macaco flavifrons*; GorGor = *Gorilla gorilla beringei*; HapGri = *Hapalemur griseus*; MacArc = *Macaca arctoides*; MacAss = *Macaca assamensis*; MacFas = *Macaca fascicularis*; MacFus = *Macaca fuscata*; MacMul = *Macaca mulatta*; MacNem = *Macaca nemestrina*; MacThi = *Macaca thibetana*; MacTon = *Macaca tonkeana*; ManSph = *Mandrillus sphinx*; PanPan = *Pan paniscus*; PanTro = *Pan troglodytes*; SemEnt = *Semnopithecus entellus*; SaiSci = *Saimiri sciureus*; VarVar = *Varecia variegata*. See Table 4 for references.

We found that, indeed, among species throughout the order of primates, female dominance increases significantly with the percentage of males in the group (Figure 2a), even when the effects of sexual dimorphism are partialled out (using the method of independent contrasts to remove effects of phylogenetic relationships, Table 2). This also holds when we confine our correlation to the genus *Macaca,* correlated over all groups and all species (Table 3).

**Table 2:** Regression analysis of female dominance, sexual dimorphism and percentage of males in the group.

	N	FemDom & SexDim	FemDom & % Males	% Males & SexDim	FemDom & % Males (SexDim partialled out)
**Raw data**					
r^2^ (slope)	22	0.269 (neg)	0.616 (pos)	0.150 (neg)	0.549 (pos)
p		0.013	<0.0001	0.075	<0.0001
**Independent contrasts**					
r^2^ (slope)	21	0.0002 (pos)	0.306 (pos)	0.038 (pos)	0.319 (pos)
p		0.678	0.002	0.631	0.001

Female dominance, sexual dimorphism and percentage of males in the group. Regression analyses (slopes and p-values) for raw data and independent contrasts branch length based on Purvis [80]. For empirical data see Table 4. FemDom = female dominance, SexDim = sexual dimorphism, % Males = percentage of males in the group.

**Table 3:** Kendall correlation between female dominance, percentage of males and sexual dimorphism.

Kendall correlation between female dominance and							
	N (groups)	*% Males*		*Sexual Dimorphism*		*% Males (SexDim partialled out)*	
		*Tau*	*Significance*	*Tau*	*Significance*	*Tau*	*Significance*
*M. mulatta*	7 ^a^	0.62	*	n.a.	n.a.	n.a.	n.a.
*M. arctoides*	4 ^b^	0.33	NS	n.a.	n.a.	n.a.	n.a.
*Macaca* (despotic^c^, all groups)	16 ^a^	0.62	**	−0.17	NS	0.62	***
*Macaca* (egalitarian^c^, all groups)	6 ^b^	0.36	NS	−0.63	NS	0.52	NS
*Macaca* (all groups)	22 ^a,b^	0.62	***	−0.01	NS	0.62	***
*Macaca* (Median per species)	8 ^a,b^	0.55	NS (p = 0.08)	0.18	NS	0.52	*

SexDim = sexual dimorphism. NS = ‘not significant’,^*^ = p<0.05, ^**^ = p<0.01, ^***^ = p<0.001 two-tailed, n.a. = not available. Difference to data set in Table 4: a = inclusion of four *M. mulatta* groups with castrated males [83], [84] and one group with ovariectomised females [85]. b = inclusion of one group of *M. arctoides* with instable hierarchy [86]. c: with *M. thibetana* re-classified as mildly despotic (grade 2) and *M. assamensis* as despotic (grade 1).

In line with the model, female dominance appears to increase significantly with the percentage of males in groups in the despotic species, *M. mulatta*, but non-significantly in the egalitarian species of *M. arctoides* (Table 3). After we had partialled out the effect of sexual dimorphism, it increases significantly among despotic groups of related species, namely of the genus *Macaca,* but among egalitarian groups this correlation is not significant (Table 3). Unexpectedly, female dominance is not correlated with sexual dimorphism; neither at a species level throughout the order of primates (using the method of independent contrasts to remove effects of phylogenetic relationships, Figure 2b, Table 2) nor at a group level in the genus *Macaca*, nor at the level of a species in this genus (Table 3). The correlation is, however, significant in the raw data of species throughout the primate order, but this is only due to lemur species.

When we excluded studies under captive conditions all results remain qualitatively the same (N = 14, data not shown).

### Discussion

The correlation between female dominance and the percentage of males is weaker in egalitarian than despotic groups both in our empirical data and in the model (Table 1). In the model, it is caused by the strong subordinance of females to males so that even with more males in the group, most of the females do not reach dominance over any of the males. Similar to these results of the model, in egalitarian groups of *M. arctoides*, female dominance is significantly lower than in despotic groups of *M. mulatta* (the median value of *M. arctoides* is 0 and of *M. mulatta* is 0.381, Mann-Whitney U test, N_1,2_ = 4,7, U = 26, P<0.024 2-tailed). Similarly, in groups of several egalitarian macaque species female dominance is significantly lower than in despotic species (median female dominance egalitarian of 0.0, despotic of 0.23; N_1,2_ = 6,16, U = 15.5, P = 0.01 two-tailed), whereas sexual dimorphism is similar for both types of groups (U = 68, P = 0.15 two tailed). Note that weaker female dominance in egalitarian groups compared to despotic ones confirms our earlier prediction based on DomWorld [11], [38], [45].

The significance of the correlation between female dominance over males and sexual dimorphism in the raw data depends on the inclusion of lemurs. In this taxon effects of sexual dimorphism may be confused with other features leading to female dominance, such as masculinised genitals [36], [46]. Use of the independent contrast method made sure that this specialised group does not exert undue influence on the correlations.

## Discussion

Our findings show that the degree of female dominance over males is associated with group composition, in accordance with the winner-loser effect (conform the self-organisation hypothesis) and hardly with sexual dimorphism (in contrast to our hypothesis based on the prior attributes). That there is so little evidence for a correlation of female dominance with sexual dimorphism is unexpected, because such a correlation is suggested by the observation that the degree of female dominance over males is high in species of the lemuriformes, where sexual dimorphism is almost absent [36], [47], low in species with intermediate sexual dimorphism, such as macaques and vervets [34], and absent in species in which sexual dimorphism is strongly male-biased, as in gorillas [48]. Therefore, in future this correlation must further be studied with more data.

Remarkably, both in the model and in empirical data, female dominance over males appears to increase with the percentage of males in the group. The explanation of this phenomenon in the model DomWorld is that a higher percentage of males in the group augments the number of interactions with high intensity. This reduces dominance of certain males relative to females, in such a way that females may be victorious over them. Furthermore, the higher proportion of interactions of females with males leads to incidental victories of females over males, and the higher intensity of these interactions also to stronger hierarchical differentiation among females. Consequently certain high ranking females may beat low ranking males and rank above them. As mentioned before, this correlation with the percentage of males in the group is not found in the model if the aggression intensity is low (in an egalitarian society), because in that case due to low hierarchical differentiation, hardly any female dominates any male. Similarly, in the genus *Macaca,* provided we partial out the effect of sexual dimorphism, this correlation with male percentage is significant among groups of despotic species, but not among groups of egalitarian ones. This confirms our earlier model-based hypothesis that the degree of female dominance is significantly lower in egalitarian primate groups than in despotic ones [11]. Since therefore, hardly any female becomes dominant over males in egalitarian societies, a correlation of female dominance with the percentage of males is impossible.

A number of alternative explanations are possible for the correlation between female dominance and group composition.

First, female dominance over males may not be a consequence of group composition, but rather its cause: perhaps females of high dominance permit the percentage of males in the group to increase, because males are non-aggressive. [This has been suggested for hyenas, 49]. However, this does not agree with our results, because in our data males are aggressive just as often as females (Wilcoxon matched-pairs signed ranks test, N = 39, W = 464.5, p<0.6642, two-tailed). Furthermore, it does not explain the process by which females become dominant over males.

Second, competition among males for access to females may become intense when females are scarce and consequently the ‘value’ of a female (and therefore her dominance over males) may increase [50]. The female ‘value’ may increase in two ways. In line with the self-organisation hypothesis it may increase through a high frequency of aggression among males for access to females (higher than in the model), which in turn leads to more frequent loser-effects among males and thus, reduces the dominance of particular males greatly, so much so that upon subsequent encounters a number of high-ranking females will be victorious and dominant over them. Besides, female dominance may increase because males allow females to win in dominance interactions, for example, in exchange for sex. If in groups with more males, more males favour females this way, female dominance may increase with the percentage of males. Such an alternative process is a kind of adaptive exchange hypothesis. This hypothesis cannot be excluded, because-even though the authors rarely state this explicitly-in a number of studies a number of females may have been in oestrous and therefore, such adaptive exchange may have happened. Further study is clearly needed.

Third, the correlation between male percentage and female dominance over males (or male subordinance to females) may be related to the effect on dominance of male residence or tenure. This effect, for instance, in rhesus monkeys [51], implies that males with a shorter residence in the group are more subordinate. Thus, to explain our positive correlation between male subordinance and the percentage of males, in groups with more males, male dominance should be lower, and therefore, male residence should be on average shorter than in groups with fewer males. However, this explanation is unlikely, because the opposite is reported for macaques: in groups with a higher percentage of males natal dispersal is lower [52] and thus, residence is probably longer.

Fourth, the correlation with male percentage may be a side-effect of support that females receive from other males or females (or both) in fights against males [53]. Also, deference of males to individual females is supposed to be displayed in order to avoid the risk of being attacked by a coalition of females [53]. That females receive support from each other against males has been described in a wide variety of species, such as lemurs, New world monkeys, such as howlers and capuchins, and Old world monkeys such as macaques, baboons, vervets, patas monkeys and several colobines [34], [53]. This occurs when males harass females or infants. We may speculate that at a higher percentage of males in the group, females and infants may more often be harassed by males. Therefore, females may also receive more often support against males [53], [54] and thus, female dominance may increase. However, due to the low frequency of coalitions compared to dyadic fights, it seems unlikely to us that coalitions alone suffice to explain the correlation of female dominance with the percentage of males in the group. Furthermore, note that, in principle, coalitions may also have self-reinforcing winner-loser effects [55], [56]. Thus, even if our correlation would (partly) be due to coalitions, the underlying process may still be the self-reinforcing winner-loser effect. Yet, causation may also operate in the opposite direction; female dominance may make it easier for females to join in coalitions against males because the risks involved are lower. In agreement with both directions of causation, female coalitions against males are more frequent in species with greater female dominance than with a weaker one, as in bonobos (co-dominance) compared to common chimpanzees (male-dominance) [31], [53], [57], [58], in despotic macaques (incidental female dominance) compared to egalitarian macaques (female subordinance), and in macaques (with incidental female dominance) compared to baboons (where females are subordinate and coalitions among females are absent) [p42, 53]. Clearly, the relationship between female dominance, coalitions and group composition needs further empirical study.

It is possible that there are other factors, still unknown, that cause females to be relatively more dominant over males in species with more males. By calculating independent contrasts between closely related species, we minimise the influence of such unknown confounding factors [59]. In addition, we have found the same positive correlation among groups of a single species, i.e. *M. mulatta*.

As to suggestions how females may benefit from a greater degree of dominance over males, it has been mentioned that (A) they may suffer less sexual coercion [53], [60], (B) they may have more freedom in choosing mates [61] [but see 62], (C) they may have more opportunity to lead group movement, which may result in feeding priority [26], [63] and (D) they may be able to protect their infants better against harassment by males [53]. On the other hand, males may suffer from a greater degree of female dominance over males because it gives them less access to females. Low rank of males prohibits them to drive away other males and to force females to mate against their will [53], [60] . From an evolutionary perspective, competition among males for females may lead to fewer males in the group [64], [65], and bigger males, thus to increased sexual dimorphism [66], and therefore, in extreme cases to stronger male dominance. However, the present study addresses only immediate effects, in which, in contrast, increased aggression with males augments female dominance over males via the winner-loser effect.

There are a number of shortcomings in our empirical data. First, the degree of sexual dimorphism in body size should be measured and correlated per group (instead of using a species-specific value). The same should be done for the degree of sexual dimorphism in intensity of aggression. Second, a systematic empirical study is needed to verify whether winner-loser effects occur in other species than rhesus monkeys [16], [17] and Japanese macaques [18]; and whether for the winner-loser effect more solid evidence can be obtained. So far evidence consists of the above random occurrence of two sequential acts of winning by the same individual and of two subsequent cases of losing (these two are indicated as ‘double initiate’ and ‘double receipt’). Third, it would be of interest to find out whether our correlation among groups of a single species is confirmed also in a number of other species besides *M mulatta.* Clearly this needs to be studied in species in which the species-specific dominance of one sex over the other is partial, *i.e*. incomplete. Thus, females should not be completely dominant already (as in certain species of lemurs), because in that case adding males may have no effect, since female dominance cannot increase any further. Neither should females be extremely subordinate to males (as is the case in gorillas), because the addition of males would still not allow females to outrank any male.

It should be noted that the model DomWorld has proved to be a useful tool for the development of integrative hypotheses based on social self-organisation [45]. However, the model is not quantitatively tuned to primate data. For instance, for an equal initial dominance of both sexes in the model, female dominance reaches at most 50%, which is below the complete female dominance of 100% described in certain primate species of the Lemuriformes. In the present model complete female dominance can only be obtained if the sexual dimorphism in initial dominance values of the sexes is reversed (representing a special adaptation in fighting power of females, for instance). Furthermore, social behaviour in the model by no means reaches the complexity of that of primates, because it neglects features such as coalitions, kin-relations and migration. We intend to add some of these features in future. Besides, the representation of dominance and of the winner-loser effect is merely phenomenological [10]. For instance, it does not separately represent a ‘bystander’ effect or ‘eavesdropping’, whereby individuals learn about the dominance of others by observing them while the others interact with third parties [67]–[69]. These effects, however, we have implicitly taken into account, because we have made individuals perceive each other's fighting capacity (as represented by their dominance value) with precision. Such precision is unlikely to be reached only by eavesdropping, memory of interactions with each opponent separately, or by direct perception of an opponent. Instead it could be obtained by using several or even all of these methods in combination, as has been suggested by empirical studies [19], [21] and theoretical ones [70], [71].

In sum, the results that are presented here demonstrate that, unexpectedly, female dominance over males in primates is not (or hardly) influenced by species-specific sexual dimorphism and that, in line with results of our model based on self-organisation, the degree of female dominance over males increases with the percentage of males in the group. In the genus *Macaca* this correlation and female dominance is stronger in despotic groups than in egalitarian ones. Our result means that, not only intra-sexual dominance relations [5], [21], but also inter-sexual dominance relations are more complex than previously thought: they are influenced by prior history and social setting. Since the winner-loser effect operates in virtually all animal species that live in groups, including humans, our results may be relevant to a broad range of taxa and be helpful also to increase our understanding of our own social system.

## Methods

### The Model

A brief summary of the model ‘DomWorld’, a model of grouping and dominance interactions, is given here [for a more complete description see 11], [12], [25], [72]. The model consists of a homogeneous, virtual world inhabited by individuals with two tendencies: grouping and performing dominance interactions. The motivation for individuals to group (whether as protection against predators or because resources are clumped) is not specified and irrelevant to the model. The same holds for the dominance interactions: They reflect competition for resources (such as food and mates), but these resources are not specified.

Individuals remain together via grouping rules. If individuals come too close, a dominance interaction may take place. The likelihood that an individual begins an aggressive interaction increases with the chance that the individual defeats its opponent. This is the so-called ‘risk-sensitive attack strategy’ [12]. The fighting power of an individual is reflected in its dominance value, i.e. DOM-value. The probability of winning of an individual over another depends both on chance and on its DOM-value relative to that of the other (i.e. its DOM-value divided by the sum of the DOM-values of both partners).

To reflect the self-reinforcing effects of victory and defeat, DOM-values are updated by increasing the DOM-value of the winner and decreasing that of the loser by the same amount. Furthermore, the size of the change in the DOM-value depends on the intensity of aggression and on the degree to which the outcome of a fight was expected: if a high ranking one wins from a low-ranking one as expected, this results in a minimal change in both DOM-values, but if unexpectedly, a low ranking one outcompetes a higher ranking individual, DOM-values of both opponents are changed by a larger amount. This method of updating makes reversals of rank possible. The change in DomValue is scaled by a factor, called StepDom, which indicates intensity of aggression (high values reflect biting, low values represent approach-retreat interactions and slapping). All else being equal, high values of StepDom imply that the change in DOM-value is greater than in the case of low values, and therefore single interactions may have a greater influence on the outcome of conflicts. Per fight the intensity of aggression is determined by its initiator.

### Experiments and the Analysis of the Model

We used the same parameters as in earlier models [11], [12], [25]. Groups consisted of 10 (or 12) individuals (which reflects the actual number of adults in many primate groups) and contain different percentages of males (for N = 10, 1–9 males). To simulate species with a different dominance style, we changed the intensity of aggression [which differs in primates between egalitarian and despotic female macaques, 43]. We compared results of groups with a high intensity of aggression, reflected in the parameter ‘StepDom’ = 1 for males (resembling despotic societies), with those of low intensity of aggression, StepDom = 0.1 for males (resembling egalitarian societies). Further, the fighting power of male primates is usually greater than in females. This is partly due to sexual dimorphism in body size and weaponry. In imitation of this, ‘females’ in the model initially have a lower dominance value than ‘males’ [initial DOM = 16 and 32, respectively, 73]. Further, because females have weaker muscles and their aggression is less intense than that of males [e.g. see 74], we studied in our model females with 10% or 80% of the StepDom-value of the males. We have conducted 40 runs per setting.

The degree of female dominance was measured as the relative position of females over males in the dominance hierarchy. It is calculated by means of the standardized Mann-Whitney-U-Value [38]: The number of males ranking below each female is counted, then the value of the statistic is computed as the sum of these counts, divided by the maximum possible value for a specific sex ratio and group size. This implies that, if in a group of 4 males and 4 females, the two highest ranking females are dominant over the two lowest ranking males, this yields a measure of female dominance over males of (2+2)/(4*4) = 0.25. Female dominance ranges from 0 (no female dominant over a male) to 1 (all females dominant over all males). Male dominance over females equals 1 minus the value of female dominance.

In our analysis we omitted the transient data by analysing data for the stable phase from period 200 to 260 (one period consists of 20 activations per individual * 10 individuals = 200 activations) [73].

### Collection and selection of empirical data

Empirical data (Table 4 and caption of Table 3) consisted of matrices of aggressive interactions or winning and were collected from the following journals up to, and including, 2006: *Animal Behaviour* (from 1965), *Behaviour* (from 1948), *American Journal of Primatology* (from 1981), *Primates* (from 1959), *International Journal of Primatology* (from 1980) and *Folia Primatologica* (from 1963). We also included unpublished data collected by Bernard Thierry (*Macaca mulatta* and *M. tonkeana*) and by Charlotte Hemelrijk and her students (*Pan troglodytes* and *Macaca mulatta*). To be used in our analysis, data on a group have to include agonistic interactions of both sexes comprising at least four adults (subadults were excluded from the analysis). We used age-categories as classified by the authors. Further, except for Table 3 (because of the small sample size), we excluded groups with medical treatments such as castration and testosterone pills and groups with an unstable hierarchy. If more groups were present per species we used the median value. We also examined data based on the ‘best study’ per species of all available groups. For the ‘best study’ we preferred groups under natural conditions to free-ranging conditions and those in free-ranging conditions to those in captivity. We chose groups that were observed for longer periods and that were closer to the natural group size as indicated by Rowe [75]. These analyses produce qualitatively similar results (not shown).

**Table 4:** Empirical Data.

Species	Condition	# Adults	% Males	SexDim	FemDom	Reference
*Alouatta palliata*	f	18	0.17	1.34	0.00	[87]
*Alouatta palliata*	f	10	0.20	1.34	0.00	[87]
*Callithrix jacchus*	n	4	0.25	0.98	0.00	[88]
*Callithrix jacchus*	n	8	0.25	0.98	0.17	[88]
*Callithrix jacchus*	n	8	0.50	0.98	0.63	[88]
*Cebus apella*	n	7	0.43	1.45	0.58	[89]
*Cercopithecus aethiops*	n	6	0.50	1.43	0.11	[90]
*Cercopithecus aethiops*	n	7	0.43	1.43	0.25	[90]
*Eulemur fulvus rufus*	n	6	0.67	0.97	0.75	[28]
*Eulemur fulvus rufus*	n	9	0.67	0.97	0.72	[28]
*Eulemur macaco flavifrons*	f	4	0.50	0.94	1.00	[91]
*Gorilla gorilla berengei*	c	6	0.17	2.24	0.00	[92]
*Hapalemur griseus*	n	4	0.50	1.09	1.00	[27]
*Hapalemur griseus*	n	5	0.40	1.09	1.00	[27]
*Macaca arctoides*	f	6	0.17	1.32	0.00	[93]
*Macaca arctoides*	c	5	0.20	1.32	0.00	[94]
*Macaca arctoides*	c	4	0.25	1.32	0.00	[95]
*Macaca assamensis*	n	23	0.48	1.64	0.21	[96]
*Macaca fascicularis*	n	16	0.56	1.59	0.83	[97]
*Macaca fascicularis*	c	8	0.13	1.59	0.00	[98]
*Macaca fascicularis*	c	10	0.10	1.59	0.00	[98]
*Macaca fuscata*	c	9	0.11	1.37	0.00	[99]
*Macaca mulatta*	c	11	0.09	1.25	0.00	[100]
*Macaca mulatta*	c	6	0.17	1.25	0.20	(Thierry, pers. comm.)
*Macaca nemestrina*	n	17	0.18	1.72	0.21	[101]
*Macaca thibetana*	n	18	0.33	1.43	0.17	[77]
*Macaca thibetana*	n	21	0.38	1.43	0.29	[77]
*Macaca thibetana*	n	18	0.44	1.43	0.39	[77]
*Macaca tonkeana*	c	8	0.13	1.21	0.00	[102]
*Macaca tonkeana*	c	13	0.23	1.21	0.20	(Thierry, pers. comm.)
*Mandrillus sphinx*	c	5	0.20	2.45	0.00	[103]
*Mandrillus sphinx*	c	9	0.22	2.45	0.00	[104]
*Pan paniscus*	c	6	0.50	1.36	0.56	[32]
*Pan troglodytes*	c	13	0.31	1.27	0.11	[105]
*Pan troglodytes*	c	15	0.33	1.27	0.09	[105]
*Saimiri sciureus*	c	6	0.17	1.18	0.20	[106]
*Semnopithecus entellus*	n	13	0.15	1.65	0.27	[107]
*Varecia variegata*	n	5	0.60	0.99	0.67	[26]
*Varecia variegata*	n	4	0.50	0.99	0.75	[26]

Condition: n = natural, f = free ranging, c = captive. SexDim = sexual dimorphism. FemDom = Female dominance, measured by the relative hierarchical position of females as the standardized Mann Whitney U statistic.

The effects of self-organisation should be clearest when we compare female dominance between groups of the same species and related species (thus omitting or reducing possibly disturbing effects of inter-specific differences). For this we used groups of species of the genus *Macaca*. Because of small sample size, we added a few groups that we excluded formerly because of medical treatment and hierarchical instability (see caption of Table 3).

### Statistical analysis of empirical data

To measure the degree of female dominance in a group, we first deduced the hierarchy of the group from a matrix of agonistic interactions. For this, we used the average of all the Dominance Indices (avDI) of an individual with all its interaction partners, which in an earlier study has been shown to be the preferred measure for this purpose [41]. The hierarchy was established by ranking individuals according to the following index: A higher average Dominance Index indicates a higher dominance position. The Dominance Index was calculated for each pair of individuals as the ratio of the number of conflicts won over a particular partner, divided by the total number of conflicts with that individual. We calculated an individual's average Dominance Index in relation to all group members, but whenever a pair did not interact at all it was excluded from the calculation of the average. The relative position of females (FemDom, Table 4) in the dominance hierarchy was calculated by means of the standardized Mann-Whitney-U-Value as explained above for the model. This calculation and that of the average Dominance Index were performed with the program Matrix Tester v223b developed by Hemelrijk and co-workers (available on request).

To perform correlations between female dominance and sex ratio for despotic and egalitarian macaque species separately, we updated the classification of female relationships of macaques by Thierry [43], [76], whereby *M. arctoides*, and *M. tonkeana* are rated as egalitarian and *M. fascicularis*, *M. fuscata*, *M. mulatta*, *M. nemestrina* as despotic. The update implies that *M. thibetana* [77] and *M. assamensis* [78] are rated as despotic (Table 3). Only for *M. mulatta* and *M. arctoides* there were sufficient groups available to perform correlations over groups within species.

For the large data set of 22 species, we used the method of independent contrasts over the median value per species. To test whether phylogenetic effects are present in our data, we used Pagel's software CONTINUOUS [79] on a composite molecular supertree of primates [80]. The maximum likelihood estimations of Lambda, which measures the degree to which the phylogeny predicts the pattern of covariance among species [79], were 0 for all parameters, indicating that phylogenetic correction would not be required for this dataset. Nevertheless, we have conducted an analysis using phylogenetically independent contrasts as proposed by Felsenstein [81]. Contrasts were generated using the program PDAP:PDTree [82]. The appropriateness of estimations of molecular branch length has been tested using the program CONTINUOUS [79]. The estimated maximum likelihood of Kappa, which stretches or compresses individual phylogenetic branch lengths [79], appeared to be 1.276 (95% confidence interval = 0.416–2.302) for all measurements (of female dominance, percentage of males and sexual dimorphism) combined. This justifies the use of estimations of molecular branch length. Because the null hypothesis of equal branch lengths was rejected (*ln*-likelihood ratio = 4.751, df = 1, p = 0.002), we reported the results of independent contrast analyses with estimations of molecular branch length. Results were, however, similar for the punctuational model (branch length = 1 for all, not shown).

To remove the effects of sexual dimorphism in body mass (SexDim, Table 1) from the relationship between female dominance and male percentage in the group (% Males, Table 1) we calculated residuals for both variables using least-squares regressions. The same procedure was followed for independent contrasts, while the regression lines were constrained to pass through the origin [82]. Besides we used a partial Kendall correlation for the limited data of groups of the genus *Macaca*.

In our study of related species of the genus *Macaca,* we did not apply the method of independent contrasts, because the number of species was limited (namely 8).
